# *Clonostachys rosea* Promotes Root Growth in Tomato by Secreting Auxin Produced through the Tryptamine Pathway

**DOI:** 10.3390/jof8111166

**Published:** 2022-11-04

**Authors:** Zhengyuan Han, Hossein Ghanizadeh, Haotian Zhang, Xinmao Li, Tiantian Li, Qi Wang, Jiayin Liu, Aoxue Wang

**Affiliations:** 1 College of Horticulture and Landscape Architecture, Northeast Agricultural University, Harbin 150038, China; 2 School of Agriculture and Environment, Massey University, Palmerston North 4442, New Zealand; 3 College of Arts and Sciences, Northeast Agricultural University, Harbin 150038, China; 4 College of Life Sciences, Northeast Agricultural University, Harbin 150038, China

**Keywords:** *Clonostachys rosea*, growth promotion, tomato, tryptophan, auxin, UHPLC-MS

## Abstract

*Clonostachys rosea* (Link) Schroers is a filamentous fungus that has been widely used for biological control, biological fermentation, biodegradation and bioenergy. In this research, we investigated the impact of this fungus on root growth in tomato and the underlying mechanisms. The results showed that *C. rosea* can promote root growth in tomato, and tryptophan enhances its growth-promoting impacts. The results also showed that tryptophan increases the abundance of metabolites in *C. rosea*, with auxin (IAA) and auxin-related metabolites representing a majority of the highly abundant metabolites in the presence of tryptophan. It was noted that *C. rosea* could metabolize tryptophan into tryptamine (TRA) and indole-3-acetaldehyde (IAAId), and these two compounds are used by *C. rosea* to produce IAA through the tryptamine (TAM) pathway, which is one of the major pathways in tryptophan-dependent IAA biosynthesis. The IAA produced is used by *C. rosea* to promote root growth in tomato. To the best of our knowledge, this is the first report on IAA biosynthesis by *C. rosea* through the TAM pathway. More research is needed to understand the molecular mechanisms underlying IAA biosynthesis in *C. rosea*, as well as to examine the ability of this fungus to boost plant development in the field.

## 1. Introduction

Fungi are ubiquitous organisms in ecosystems, and some of them play a crucial role in helping plants grow better, absorb nutrient, tolerate stress and resist pathogens [1]. Several studies have also shown that fungi can promote plant growth. For example, *Trichoderma virens* can promote growth in *Arabidopsis thaliana* (L.) Heynh. through stimulating the expression of genes associated with the indole-3-acetic acid (IAA) pathways [2]. In addition, *Colletotrichum tofieldiae* has been shown to enhance the growth of plants under phosphorus-deficient conditions [3], and *Aspergillus fumigatus* (HK-5-2) can promote the growth of soybean (*Glycine max* L.) [4].

*Clonostachys rosea*, a member of the family Bionectriaceae, is a filamentous fungus that is widely distributed in soils [5]. *Clonostachys rosea* has been widely used for biological control, biological fermentation, biodegradation and bioenergy [5,6]. *Clonostachys rosea* can also improve plant growth. For instance, it has been shown that *C. rosea* promotes the growth of tomato plants [6,7] and boosts root development in lettuce grown hydroponically [8].

The relationship between plant and fungi can be mutually beneficial. Plant roots exude nutrients such as sugar and amino acids, which will be used by fungi; in return, fungi produce metabolites to improve plant growth [9]. These metabolites can act as signal transduction factors to stimulate the expression of pathways corresponding to plant growth. For instance, it has been shown that *T. harzianum* can promote growth in maize (*Zea mays* L.) through enhancing the expression of genes associated with auxin biosynthesis [10]. Some of the metabolites can bolster plant immune systems and plant health. For instance, metabolites such as salicylic acid and jasmonic acid produced by beneficial fungi can promote plant health through regulating the pathways enhancing plant immunity against diseases [11].

Auxin (IAA) is a prominent growth-promoting hormone in plants [12]. The biosynthesis pathway of IAA is divided into the tryptophan-dependent synthesis pathway and the tryptophan-independent synthesis pathway [13]. In the tryptophan-dependent pathway, which is the primary pathway for IAA biosynthesis, tryptophan is used as a precursor for the synthesis of auxin [13]. In fungi, tryptophan is converted to indole-3-acetamide (IAM) by tryptophan-2-monooxygenase (iaaM), and IAM is subsequently catalyzed to IAA by IAM-hydrolase (iaaH) [14].

In our preliminary experiment, we noted that *C. rosea* promoted root growth in tomato seedlings. To the best of our knowledge, the promoting impacts of this fungus on tomato root growth have not been reported. In this research, we explore the mechanisms associated with root growth promotion in tomato plants by *C. rosea* using an ultrahigh-performance liquid chromatography–tandem mass spectrometry (UHPLC-MS/MS) approach. This technique uses the advantages of the high separation ability of liquid chromatography and the excellent quantitative and qualitative ability of mass spectrometry to detect trace levels of substances in microbial metabolites [15]. The results of this research provide prerequisite information for the development of strategies towards using *C. rosea* as inoculants for improving plant growth.

## 2. Materials and Methods

### 2.1. Materials and Reagents

Acetonitrile (≥99.99%), methanol (≥99.99%) and glucose were purchased from Tianjin Kemiou Chemical Reagent Co., Ltd. (Tianjin, China). Agar was purchased from Coolaber Co., Ltd. (Beijing, China). L-tryptophan was purchased from Shanghai Macklin Biochemical Co., Ltd. (Shanghai, China). Ethyl acetate (≥99.7%) was purchased from Tian Jin FuYu Chemical Co., Ltd. (Tianjin, China). Acetonitrile and methanol were of HPLC grade; all other reagents were of analytical grade.

### 2.2. Culture Conditions

*Clonostachys rosea* (WY-1) was isolated from soil samples using the method described previously [7]. The isolated fungus was cultured in either a liquid potato dextrose medium (CR) or a liquid potato dextrose medium containing L-tryptophan (CR + L). The CR group culture medium consisted of 20 g of glucose, 1000 mL of distilled water, and 200 g of filtrate of boiled potatoes. The CR + L group culture medium consisted of 20 g of glucose, 1000 mL of distilled water, 200 g of filtrate of boiled potatoes, and 20 g of L-tryptophan. The cultured media were maintained in an incubator (ZQZY-85BN, Zhichu, Shanghai, China) at 30 °C and 200 r min^−1^ for 7 days.

### 2.3. Tomato Root Growth Experiment

In this research, we initially ran a preliminary experiment (Experiment 1) to assess the effect of fermented broth of *C. rosea* (10^6^ cfu mL^−1^) on root growth in tomato plants. For this, tomato seeds were disinfected with 75% ethanol for 20 s and then soaked in 3% H_2_O_2_ solution for 20 min. The disinfected seeds were then soaked in distilled water for 6 h. Subsequently, 20 tomato seeds were soaked in the fermentation broth of *C. rosea* for 6 h (treatment group). To exclude the effect of culture medium components (i.e., glucose and potato extract) on root growth, 20 seeds were soaked in the filtrate of the culture medium (i.e., liquid potato dextrose) for 6 h (PD). For the control group, 20 seeds were soaked in distilled water for 6 h (CK). Seeds from all treatment groups were placed in germination papers soaked with distilled water and the papers were maintained vertically in an incubator at 25 °C and a photon flux density of 800 μmol m^−2^ s^−1^ for 7 days. In a different experiment (Experiment 2), the effects of *C. rosea* grown on liquid potato dextrose (CR) and *C. rosea* grown on liquid potato dextrose plus tryptophan (CR + L) on the root development of tomato seeds were investigated. For this, tomato seeds were disinfected using the method mentioned above, and then placed in Petri dishes containing either CR or CR + L medium, with 10 seeds in each Petri dish. The Petri dishes were maintained under the same conditions outlined above, and seedlings were assessed after 3 days. All experiments were conducted in a completely randomized design with three replicates.

### 2.4. Plant Experiments

Tomato seeds were disinfected with 3% H_2_O_2_ for 20 min and then sown in pots containing sterilized medium as described previously [6]. Pots were kept in a greenhouse under 16 h light/8 h dark, at 25 °C and relative humidity of 60%. This experiment consisted of three treatment groups, namely CR, CR + L and sterile water (control). Tomato seedlings were irrigated with 100 mL of water of the solution containing either CR or CR + L when they were at the 6-leaf stage. The experiment was conducted in a completely randomized design with three replicates.

### 2.5. Metabolite Extraction

In this part of the research, the metabolic profile for *C. rosea* grown in CR and CR + L treatment groups was investigated. For this, 100 mL of the mixture was taken from each group and placed in an ultrasonic cell homogenizer (SCIENTZ-IID, SCIENTZ, Ningbo, China) for 30 min (180 W work, 5 s; rest, 10 s). The homogenized mixtures were centrifuged (3K15, SIGMA, Yangzhou, China) at 4 °C and 10,000 r min^−1^ for 10 min, and the supernatants were collected. The supernatants were extracted with 100 mL of ethyl acetate (0.5% *v*/*v*) three times. Ethyl acetate extracts were collected using a liquid separation funnel, concentrated to 1 mL by a rotary evaporator, and then filtered through a 0.22 µm filter three times [16].

### 2.6. UHPLC-MS/MS Analysis

In this study, a UHPLC-MS (AB SCIEX TRIPIETOF 5600, AB Sciex, Framingham, MA, USA) approach was used to isolate and identify the metabolites from CR and CR + L treatment groups using a ACQUITY UHPLC BEH C18 chromatographic column (100 mm × 2.1 mm, 1.7 μm) at 30 °C and a detection wavelength of 512 nm. For each run, a sample volume of 5 μL was injected and analyzed for 25 min at a mobile phase flow rate of 0.4 mL min^−1^. The mobile phase A was an aqueous solution containing 0.1% formic acid, and the mobile phase B was pure acetonitrile. The gradient elution program was as follows: 0–5 min phase A, 5–10% mobile phase B; 5–10 min, 10–15% mobile phase B; 10–15 min, 15–95% mobile phase B; 15–20 min, 95% mobile phase B; 20–20.1 min, 95–5% mobile phase B; and 20.1–25 min, 5% mobile phase B. Positive and negative ionization modes were used, and the scanning range for primary mass spectrometry and secondary mass spectrometry was 100–1000 *m*/*z*. Nitrogen was used in each gas channel. Mass spectrometric conditions are summarized in Table S1. The final experimental results were analyzed by PeakView 2.2 and MS-DIAL V.4.90 software.

### 2.7. Statistical Analyses

The data from experiment 1 were analyzed using a protected pairwise *t*-test. The data from Experiment 2 and plant experiment were subjected to one-way analysis of variance (ANOVA) and means were separated using Tukey’s test at 5% level of probability. All statistical analyses were performed using GraphPad Prism 9.0. All experiment were repeated three times.

## 3. Results

**Figure 1:**
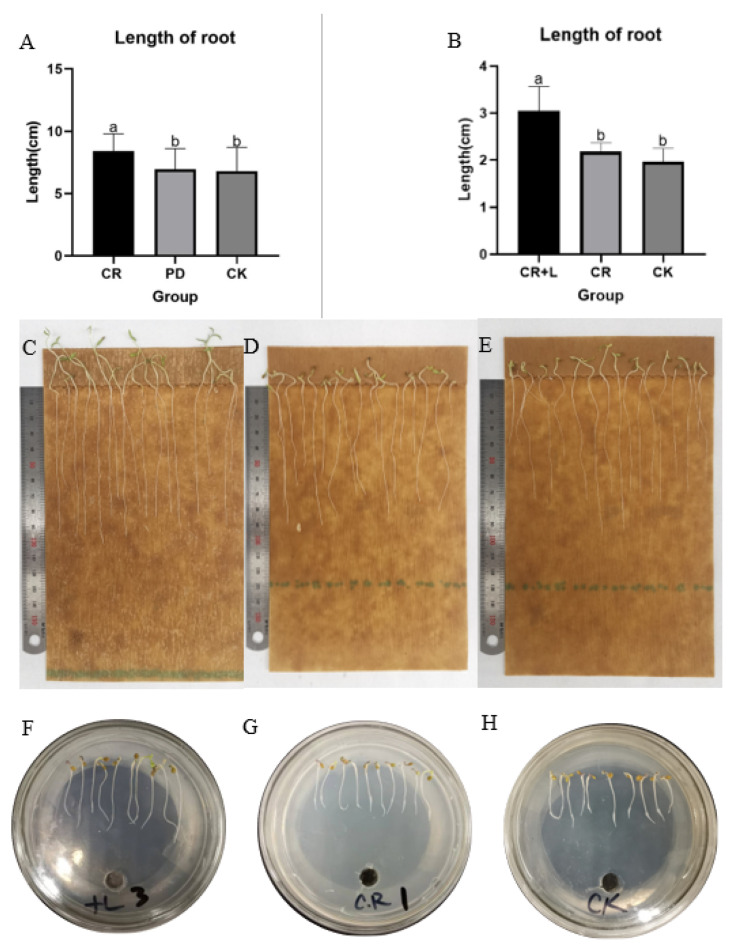
The growth of tomato seedlings in Experiment 1 (**A**,**C**–**E**) and Experiment 2 (**B**,**E**,**G**,**H**). (**A**) Statistical comparison between seedlings treated with fermented broth of *C. rosea* (CR), seedlings treated with culture medium (PD) and seedlings treated with sterile water (CK). (**B**) Statistical comparison between seedlings treated with CR, seedlings treated with *C. rosea* plus tryptophan (CR + L) and seedlings treated with CK. (**C**) Seedlings treated with CR, (**D**) seedings treated with PD, (**E**) seedlings treated with CK, (**F**) seedlings treated with CR + L, (**G**) seedlings treated with CR and (**H**) seedlings treated with CK. Within panels (**A**,**B**), bars that do not share a similar letter are significantly different at 5% probability according to Tukey’s test.

**Figure 2:**
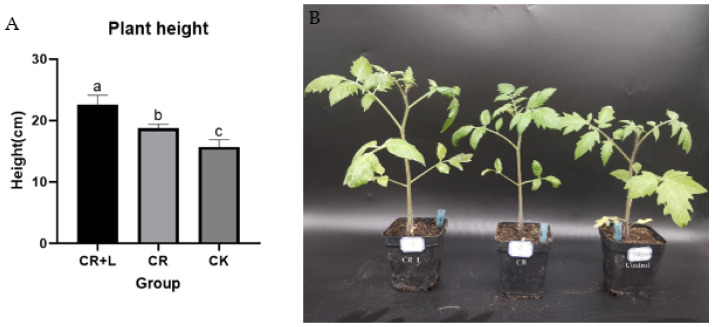
The effect of *C. rosea* (CR), *C. rosea* plus tryptophan (CR + L) and sterile water (CK) on tomato plants. (**A**) Statistical comparison between plants treated with CR, plants treated with CR + L and plants treated with CK. Bars that do not share a similar letter are significantly different at 5% probability according to Tukey’s test. (**B**) The effect of CR, CR + L and CK on tomato plants. The photograph was taken at 7 days after treatment.

The results from Experiment 1 reveal that tomato seeds treated with the fermented broth of *C. rosea* (treatment group) had a significantly longer root length compared to those treated with sterile water (CK) and filtrate of culture medium (PD) (Figure 1A,C–E). The results also show that the average root length of the tomato seedlings in the treatment group was 8.41 cm, while the average root length of tomato seedlings in the CK and PD groups was 6.81 and 6.94 cm, respectively. This result suggests that treatment with *C. rosea* increased the root length by almost 20% in tomato seedlings, and that the culture medium had no impact on the root growth. In Experiment 2, the response of tomato seedlings was assessed and compared among *C. rosea* (CR), *C. rosea* plus tryptophan (CR + L) and control (CK), and the results show that tomato seedlings treated with CR + L had longer root length than the other treatments (Figure 1B,E,G,H). Both CR and CR + L treatments also promoted the growth of tomato plants when they were applied after to the roots at the six-leaf growth stage (Figure 2). However, it appears that the CR + L group had greater positive impacts on tomato growth than the CR group, as the plants in this treatment group grew bigger than those in the CR group. In addition, it appears that tryptophan addition promoted the positive impact of the fungus as it accelerated the rate of root growth in the treated seedlings, with CR + L treated seedling growing longer roots than those treated with CR after 3 days. Taken together, the results from all three experiments suggest that *C. rosea* can promote growth in tomato plants, and tryptophan can enhance the growth-promoting effects of this fungus.

**Figure 3:**
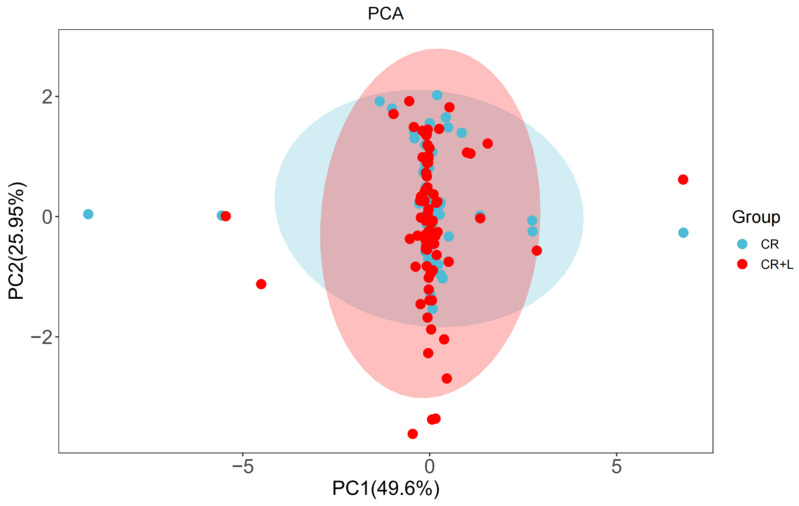
Principal component analysis (PCA) of metabolite profiles in the *C. rosea* plus tryptophan (CR + L) and *C. rosea* (CR) groups. The red and blue dots represent the metabolites in the CR + L and CR groups, respectively. The red and blue ellipses correspond to the metabolite profiles of CR + L and CR groups, respectively.

To examine the metabolic profiles of *C. rosea* and the impact of tryptophan on metabolites, the metabolites in CR and CR + L treatment groups were analyzed and identified by UHPLC-MS (Figure 3 and Figure S1). The analyses were performed in both positive and negative ionization modes. In the positive ionization mode, 267 and 316 effective substances were identified in the CR and CR + L groups, respectively. In the negative ionization mode, 95 and 209 substances were identified in the CR and CR + L groups, respectively. These results indicate that in both detection modes, a higher abundance of metabolites was produced in the CR + L group than the CR group; thus, tryptophan altered the metabolic profile of *C. rosea*.

A principal component analysis of metabolite associated with CR + L and CR groups revealed that the first two principal components (PCs) can explain 75.6% of the variation (Figure 3). The PCA plot shows that the metabolite richness of the CR + L group was higher than that of the CR group. Although, there was no clear separation between the metabolites of both groups, there were several metabolites that differed between both groups, indicating that tryptophan affected the metabolite profiles of *C. rosea.*

**Figure 4:**
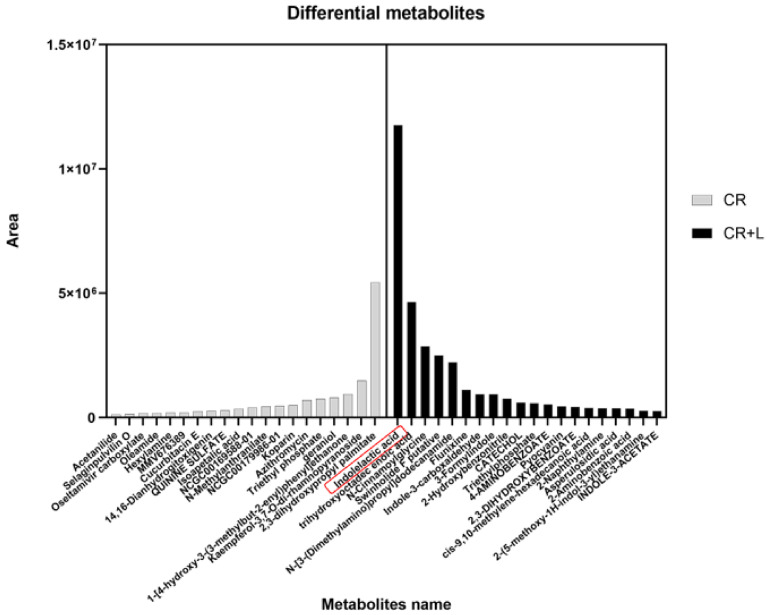
The top 20 metabolites detected by UHPLC-MS in the *C. rosea* (CR) and *C. rosea* plus tryptophan (CR + L) groups.

To study whether these differential metabolites led to a growth-promoting effect, we distinguished and classified the metabolites into two groups based on the treatments (CR vs. CR + L), and selected the top 20 differentially abundant metabolites (Figure 4). The results showed that the greatest differentially produced metabolite in the CR group was 2,3-dihydroxypropyl palmitate, while the greatest differentially produced metabolite in the CR + L group was IAA. In addition, we recorded a large number of auxin-related substances, such as tryptamine (TRA) and indole-3-acetaldehyde (IAAId) for the CR + L group. The greater abundance of the above-mentioned auxin-related substances in the CR + L group may imply that exogenous tryptophan has increased the abundance of IAA through stimulating an auxin biosynthesis pathway that utilizes TRA and IAAId.

**Figure 5:**
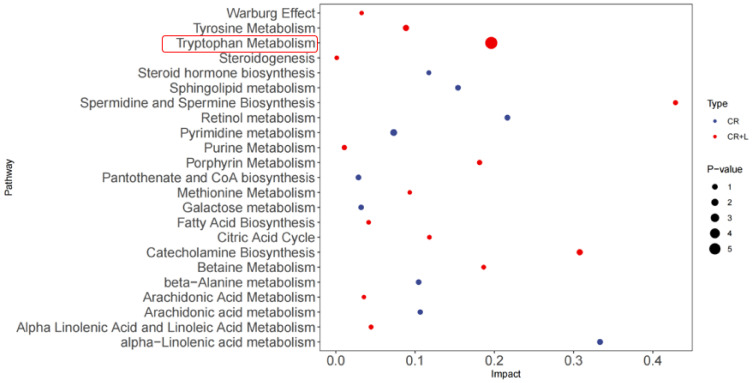
Differential metabolic pathways in the *C. rosea* (CR) and *C. rosea* plus tryptophan (CR + L) groups. Red circles represent the differential metabolic pathways in the CR group. Blue circles represent the differential metabolic pathways in the CR + L group. The size of the circle represents the *p* value.

**Figure 6:**
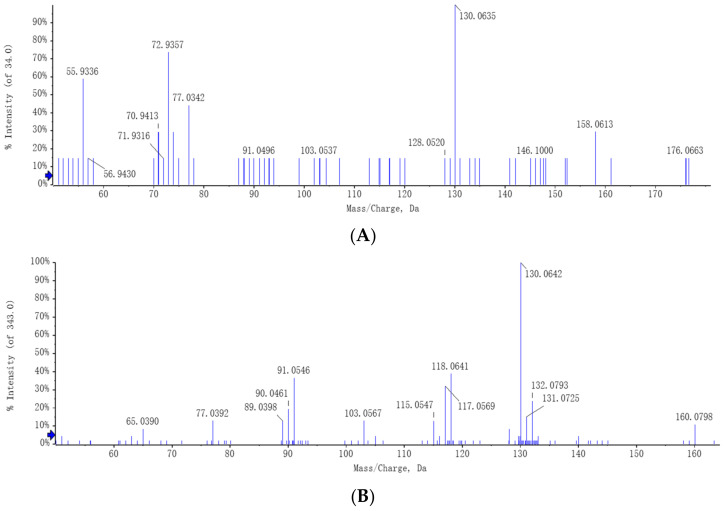

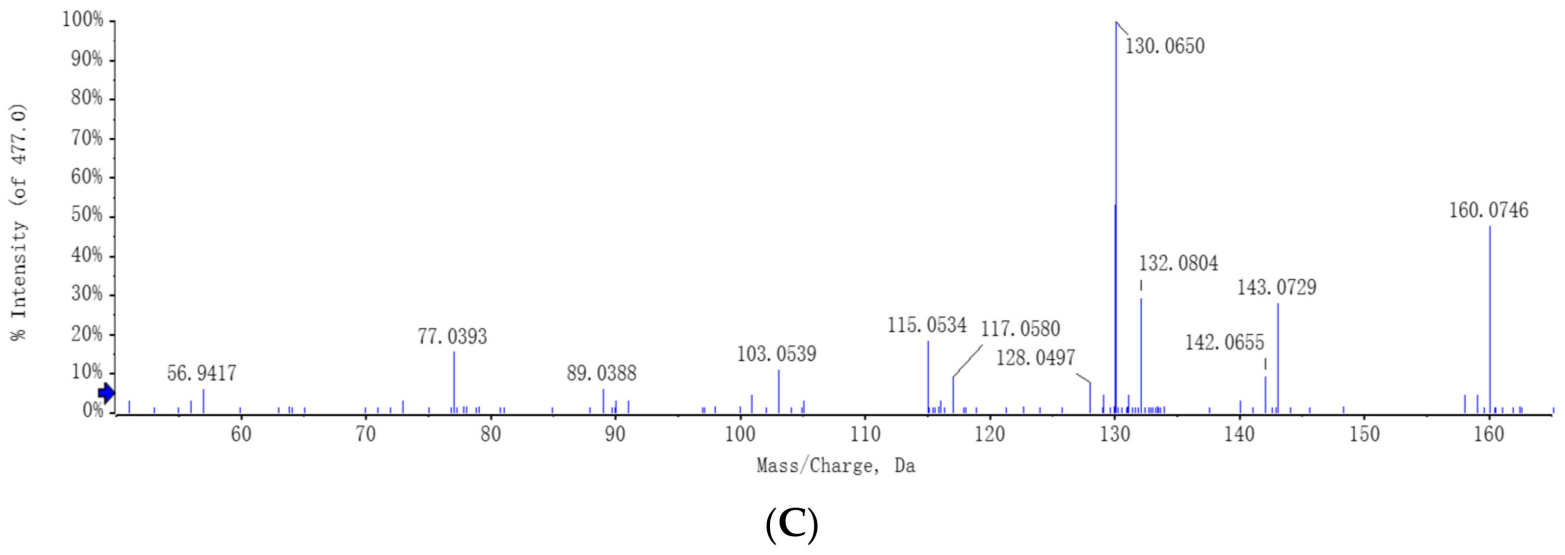
The metabolites related to auxin biosynthesis in the *C. rosea* plus tryptophan (CR + L) group determined by UHPLC-MS. (**A**) MS/MS spectrum of auxin (IAA), (**B**) MS/MS spectrum of indole-3-acetaldehyde (IAAId), (**C**) MS/MS spectrum of tryptamine (TRA). Determinations were performed from at least three independent samples.

It also appears that exogenously applied tryptophan altered the metabolic profile of *C. rosea* in the CR + L group (Figure 5). The results showed that exogenous tryptophan greatly changed the pathways corresponding to tryptophan metabolism, indicating that *C. rosea* has the ability to utilize tryptophan and can convert it into the auxin analogs and IAA. The full-scan and MS spectra of auxin and auxin analogs in the CR + L group are shown in Figure 6. The MS spectrum revealed a molecular ion at *m*/*z* 130.06 representing IAA (Figure 6A). The MS spectrum also showed molecular ions at *m*/*z* 143.07 and 160.07 representing IAAId and TRA, respectively (Figure 6B,C).

## 4. Discussion

In nature, there is a consistent interaction between microorganisms and plants, with fungi being one of the most common organisms that interact with plants [17]. Some fungi promote plant growth and antagonize pathogens, so they have great potential in agricultural production [18]. *Clonostachys rosea* can be used as a biological control agent to antagonize *Botrytis cinerea* through the stimulation of protective substances and enzyme activities in plants [6]. This fungus can also parasitize plants and utilize nutrients provided by plants; in return, it enhances the growth of the host plant [5]. However, the mechanisms underlying the growth-promoting impacts of *C. rosea* in plants have not been completely elucidated.

In this research, we intended to shed light on some of the aspects of growth-promoting mechanisms of *C. rosea* when it is applied to tomato plants. The results showed that *C. rosea* caused a strong stimulation of root growth in tomato seedlings. In addition, when *C. rosea* was applied to the roots, it stimulated the growth of above-ground foliage of tomato plants. However, when *C. rosea* was used in conjunction with tryptophan, greater growth-promoting impacts were recorded in the treated seedlings compared to those treated with *C. rosea* only. These results suggested that tryptophan enhances the mechanism underlying the growth-promoting impact of *C. rosea*, and that this fungus uses tryptophan to synthesize growth-stimulating metabolites.

To examine this hypothesis, we used a UHPLC-MS approach to explore and compare the specific structures of growth-promoting metabolites after *C. rosea* and the *C. rosea* plus tryptophan treatment. We also aimed to understand if tryptophan addition would affect the metabolic profile of *C. rosea*, and gain insight into the physiological function corresponding to changes in metabolic profiles in *C. rosea* after tryptophan addition. The results showed that the content of metabolites was greater in the tryptophan-treated *C. rosea*, with IAA and auxin-related compounds comprising of a majority of the highly abundant metabolites. This result implies that tryptophan addition changed the metabolic pathway in *C. rosea*. In addition, MS spectrum identified IAAId and TRA in the tryptophan-treated *C. rosea* group, which are metabolites of tryptophan [19], indicating that *C. rosea* converted tryptophan into both metabolites. As both IAAId and TRA are precursors of IAA, the result from MS analysis imply that *C. rosea* produced IAA in the presence of tryptophan.

Phytohormones, such as auxin, cytokinins and gibberellins, are involved in several aspects of plant growth [20]. These phytohormones can be produced by plants to maintain normal plant growth [21]. Several microorganisms engaged in plant–microbe interactions, including fungi, have been shown to produce IAA to improve plant growth [22]. Auxin is known to be responsible for the initiation and growth of lateral roots formed in the post-embryonic stage [23]. Given the direct role of auxin in root growth, and the results of this research showed that adding tryptophan to *C. rosea* culture further enhanced its impacts on root growth in tomato plants, we suggest that this fungus exerts its plant growth-promoting effect through IAA secretion. In agreement with our results, it has been shown that a root-endophytic fungus CJAN1179 from *Cymbopogon jwarancusa* (Jones)Schult. enhanced root formation and growth in *Arabidopsis thaliana* in the presence of tryptophan, through secreting IAA using tryptophan as a precursor [24].

**Figure 7:**
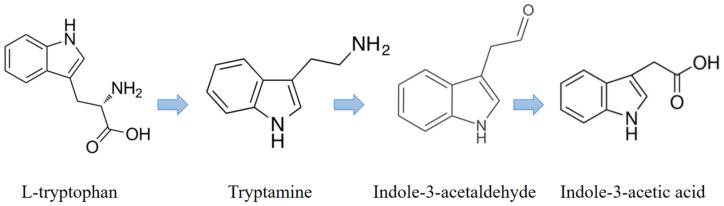
The tryptamine (TAM) pathway used by *C. rosea* to produce IAA using tryptophan as a precursor.

The tryptophan-dependent pathway is the primary pathway for IAA biosynthesis in fungi where tryptophan is used as the primary precursor for IAA biosynthesis. The IAA biosynthesized by fungi is used as a communication signal between the fungi and the host plant roots [25]. Additionally, it is known that the IAA synthesized by fungi can affect gene expression in the target organism [26]. Our results show that *C. rosea* produces IAA along with two important tryptophan metabolites, namely IAAId and TRA, which are involved in the tryptamine (TAM) pathway. The TAM pathway is one of the major pathways in tryptophan-dependent IAA biosynthesis. The results of this research suggest that *C. rosea* used the TAM pathway to synthesize IAA (Figure 7 and Figure S2), and the synthesized IAA promoted root growth in tomato plants (Figure S3).

## 5. Conclusions

In this research, we demonstrated that *C. rosea* can promote growth in tomato plants through enhancing root growth. The results also showed that *C. rosea* is capable of producing IAA in the presence of tryptophan through the tryptophan-dependent TAM pathway. The mechanism of IAA production is known only for very few fungi; to the best of our knowledge, this is the first report on IAA biosynthesis by *C. rosea* through the TAM pathway. More research is needed to understand the molecular mechanisms underlying IAA biosynthesis in *C. rosea*, as well as to examine the ability of this fungus to boost plant development in the field. Information gleaned from such studies can aid to explore strategies towards using *C. rosea* as inoculants for improving the growth of desirable plants.

## Data Availability

Data are contained within the article or Supplementary Materials.

## Supplementary Materials

The following supporting information can be downloaded at: https://www.mdpi.com/article/10.3390/jof8111166/s1, Table S1:Mass spectrometric conditions, Figure S1: Metabolic profile of the CR and CR + L groups detected by UPLCMS in positive and negative detection modes. Figure S2: biosynthesis. The part of red showed the biosynthesis of *Clonostachys rosea*, Figure S3: Graphical abstract demonstrating the positive impact of *Clonostachys rosea* plus tryptophan on tomato growth.
