# Supplementary material for: Clonostachys rosea Promotes Root Growth in Tomato by Secreting Auxin Produced through the Tryptamine Pathway
Source: J Fungi (Basel). 2022 Nov 4;8(11):1166. doi: 10.3390/jof8111166 (PMC9695606; doi:10.3390/jof8111166)
Supplement: Supplementary file 1 [file jof-08-01166-s001.zip › jof-1990227-supplementary.pdf]

Supporting information

Table S1 Mass spectrometric conditions

Parameter	ESI-MS	ESI-MS
Scan Type	TOF	TOF
Ion Spray Voltage Floating (ISVF)	5500 V	5500 V
Source Gas 1 (GS1)	50 psi	50 psi
Source Gas 2 (GS2)	50 psi	50 psi
Curtain Gas (CUR)	35 psi	35 psi
Temperature (TEM)	550 °C	550 °C
Declustering Potential (DP)	90 V	90 V
Collision Energy (CE)	10 V	35 V
Collision Energy Spread (CES)		15 V

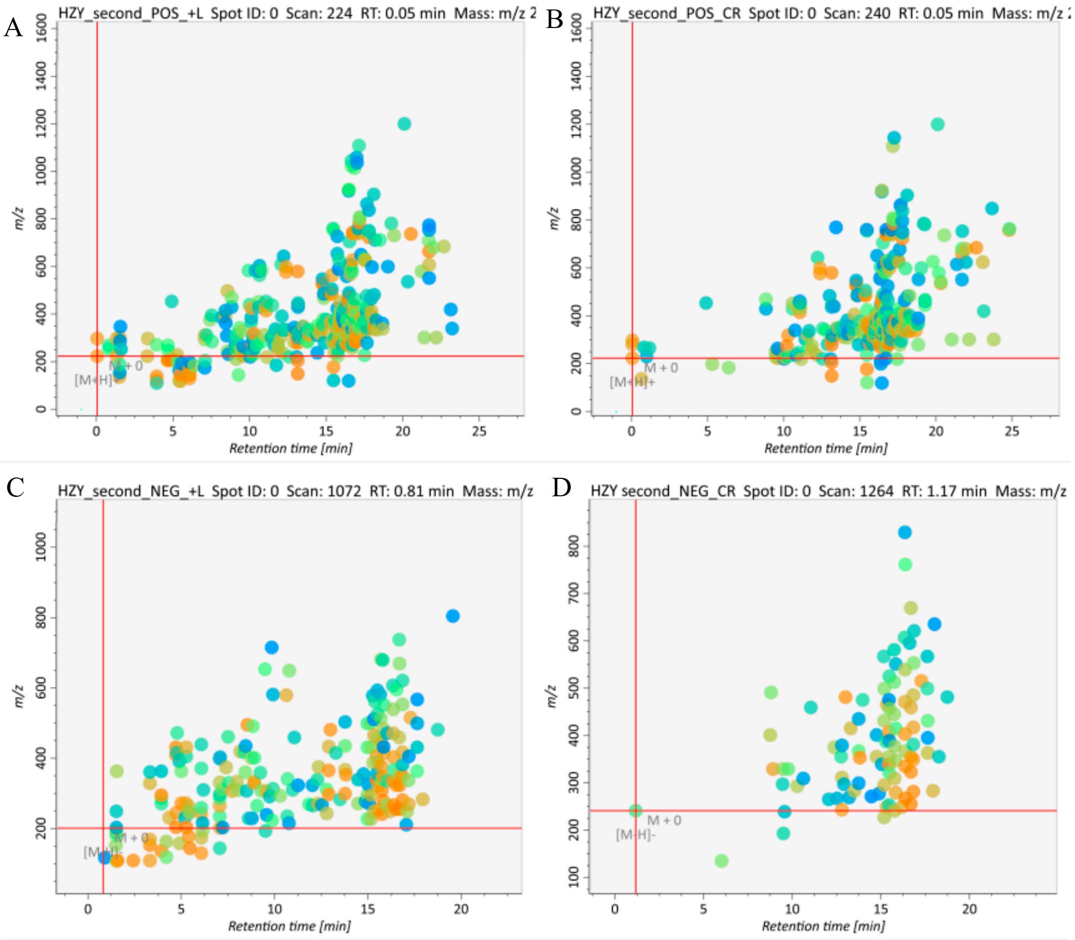

Figure S1. Metabolic profile of the CR and CR + L groups detected by UPLC–MS in positive and negative detection modes. A) metabolites of the CR + L group in the positive ionization mode. B) metabolites of the CR group in the positive ionization mode. C) metabolites of the CR + L group in the negative ionization mode. D) metabolites of the CR group in the negative ionization mode.

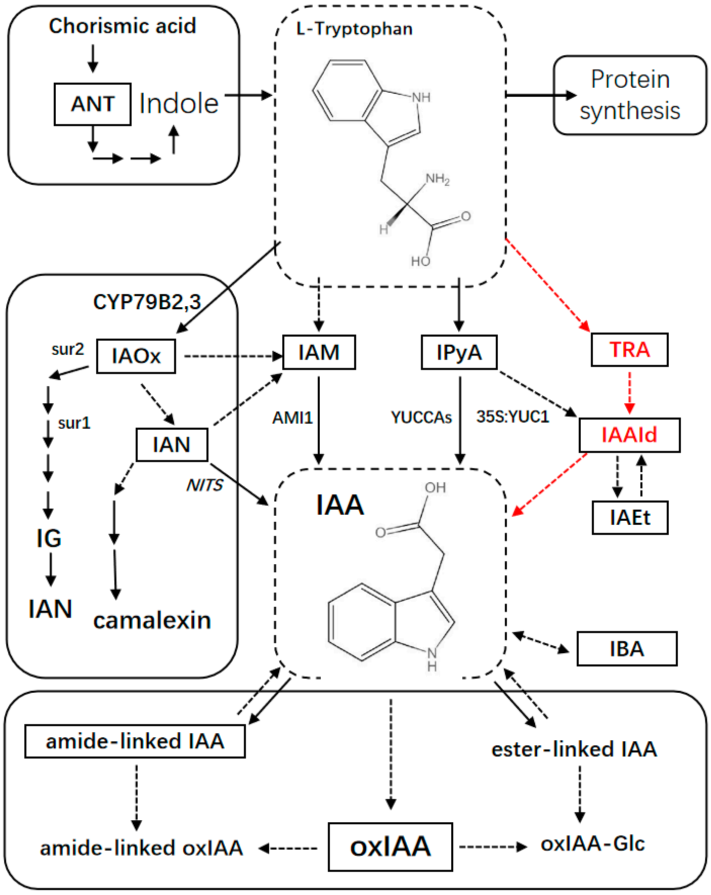

Figure S2. biosynthesis. The part of red showed the biosynthesis of *Clonostachys rosea.*

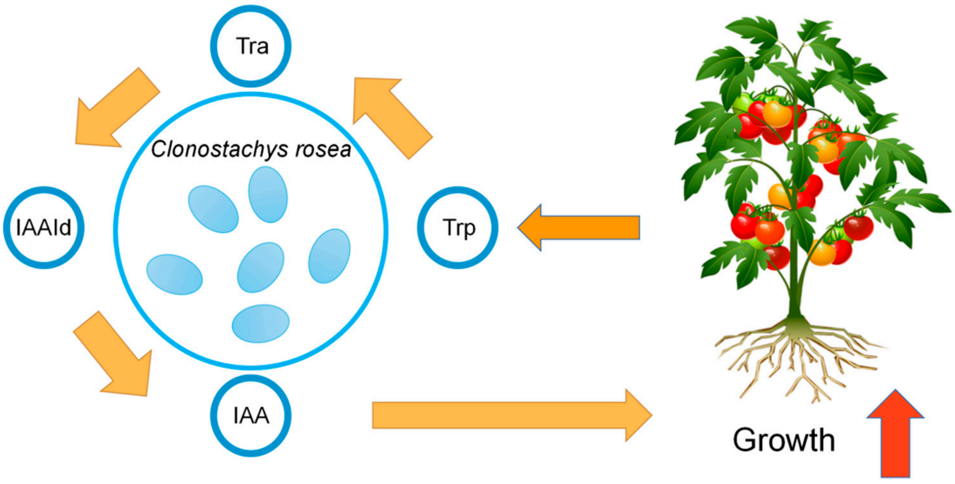

Figure S3. Graphical abstract demonstrating the positive impact of *Clonostachys rosea* plus tryptophan on tomato growth.
